# Navigating the redox landscape: reactive oxygen species in regulation of cell cycle

**DOI:** 10.1080/13510002.2024.2371173

**Published:** 2024-07-07

**Authors:** Viktoria Mackova, Martina Raudenska, Hana Holcova Polanska, Milan Jakubek, Michal Masarik

**Affiliations:** a Department of Pathological Physiology, Faculty of Medicine, Masaryk University, Brno, Czech Republic; b Department of Physiology, Faculty of Medicine, Masaryk University, Brno, Czech Republic; c BIOCEV, First Faculty of Medicine, Charles University, Vestec, Czech Republic; d Institute of Pathophysiology, First Faculty of Medicine, Charles University, Prague, Czech Republic

**Keywords:** Cell cycle, reactive oxygen species, oxidative stress, proliferation, redox state, redox-sensitive targets, cell cycle signaling

## Abstract

**Objectives:** To advance our knowledge of disease mechanisms and therapeutic options, understanding cell cycle regulation is critical. Recent research has highlighted the importance of reactive oxygen species (ROS) in cell cycle regulation. Although excessive ROS levels can lead to age-related pathologies, ROS also play an essential role in normal cellular functions. Many cell cycle regulatory proteins are affected by their redox status, but the precise mechanisms and conditions under which ROS promote or inhibit cell proliferation are not fully understood.

**Methods:** This review presents data from the scientific literature and publicly available databases on changes in redox state during the cell cycle and their effects on key regulatory proteins.

**Results:** We identified redox-sensitive targets within the cell cycle machinery and analysed different effects of ROS (type, concentration, duration of exposure) on cell cycle phases. For example, moderate levels of ROS can promote cell proliferation by activating signalling pathways involved in cell cycle progression, whereas excessive ROS levels can induce DNA damage and trigger cell cycle arrest or cell death.

**Discussion:** Our findings encourage future research focused on identifying redox-sensitive targets in the cell cycle machinery, potentially leading to new treatments for diseases with dysregulated cell proliferation.

## Introduction

Reactive oxygen species (ROS) are chemically reactive compounds generated as by-products of cellular metabolism and oxygen consumption. Far from being expendable metabolic derivatives, they are significant signaling molecules in various physiological and pathological processes. Cells produce ROS in response to various stimuli, including growth factors, cytokines, and stressors. ROS comprise free radicals such as superoxide anion ($O_{2}^{∙-}$) and hydroxyl radical (${}^{∙}\;\mathrm{OH}$), as well as non-radical species like hydrogen peroxide (H_2_O_2_) and singlet oxygen (^1^O_2_). The capacity of cellular oxidants to function as signaling molecules can be influenced by their potency. For instance, potent oxidants like ozone and nitrogen dioxide can non-selectively oxidize molecules, leading to irreversible pathophysiological reactions and loss of protein function [1]. Conversely, physiological endogenously produced oxidants, such as controlled amounts of H_2_O_2_, often serve as specific and reversible signal transducers, providing additional regulatory mechanisms [1].

Determining the precise threshold of exogenous oxidants applied to cells that leads to proliferation, oxidative stress, or apoptosis currently presents a significant challenge. Experimental investigations utilizing H_2_O_2_ often yield divergent outcomes with similar concentrations or analogous outcomes with different concentrations (Table 1). For instance, studies with NIH3T3 fibroblasts demonstrate that treatment with low H_2_O_2_ concentrations (0.02–0.13 µM) induces cell proliferation, while higher concentrations (0.25–2 µM) lead to cell death [2,3]. The oxidative damage and slower blastocyst formation have been detected in mouse zygotes at concentrations of 0.03 mM H_2_O_2_ [4], and Martínez Munõz et al. [5] even utilized concentrations as high as 3–5 mM H_2_O_2_ to induce oxidative stress without significant cell lysis in cell culture. Chang et al. [6] synthesized findings from diverse studies, proposing that concentrations ranging from 1 to 15 µM signal proliferation, while the range of 100–200 µM halts the cell cycle, and concentrations spanning 0.5–5 mM induce apoptosis or necrosis [6]. However, further research is still needed to accurately determine the most critical variables in the experimental application of H_2_O_2_ to induce and measure the effects of oxidative stress. The methodology of H_2_O_2_ dilution preparation may be one of the significant variables. Evidence suggests that dilution in the cell culture medium can cause H_2_O_2_ to interact with its components, influencing its final concentration [5,7]. Moreover, Ransy et al. [8] pointed out that cellular catalase rapidly converts most of the applied H_2_O_2_ into oxygen within a few minutes [8]. A high concentration of oxygen in the cell may increase the probability of the formation of singlet oxygen, contributing to oxidative damage [8]. Therefore it is essential to better consider and understand the process preceding oxidative stress to prevent misidentification of molecular targets and mistakes in experimental interpretation. Other influential factors may include incubation time, cell line type, and the cell cycle phase during treatment.

**Table 1.** Summary of experimental outcomes and conditions used in H_2_O_2_ treatments across various cell lines and organisms.

Cell line	Organism	H_2_O_2_ concentration	Treatment duration	Described effect on cells	Reference	Detection method	H_2_O_2_ solvent	
oral keratinocytes	human	10–1000 μM	15 min–8 h	> 90% of cells were viable	[9]	MTT assay	culture medium	
hepG2 cells	human	25–800 μM	4–24 h	IC50 value of 371.3 uM. Below 4 h of incubation with 400 uM H2O2, cell viability showed no significant change	[10]	MTT assay	culture medium	
IMR-90 (fibroblasts)	human	50-350 μM	2 h	cytotoxic effects of concentrations above 200 uM, all cells killed without activation of caspase-3 after treatment above 300 uM, dose-dependent caspase activation after 16 h in cells treated with 50–200uM, a large proportion of cells treated with 150uM growth arrested and senescent, 150uM – growth arrest and apoptosis	[11]	flow cytometry, immunoblotting	culture medium	
NIH3T3 fibroblasts	mouse	200–500 μM	16 h	± 50–80% cell death	[12]	flow cytometry	not specified	
lens epithelial cells	human	50–100 μM	4 h	proteasome activity inactivated by 50–80%	[13]	proteasome peptidase activity assay	H_2_O_2_ is produced through incubating cells with glucose oxidase (20 or 40 mU/ml) in a serum-, pyruvate-, and phenol red-free medium, supplemented with D-glucose (4500 mg/L).	
HeLa cells	human	1–10 000 μM	10 min	several MPKs are inhibited through oxidation of catalytic cysteine, sustained JNK activation	[14]	Western blot (WB)	not specified	
fetal neurospheres	human and murine	2–4 μM	not specified	positive effect on multipotent neurosphere formation	[15]	FACS	culture medium	
mammary adenocarcinoma	mouse	1–1 000 μM	15 min	partially reversible inhibition of cell attachment to laminin and fibronectin in a dose-dependent manner	[16]	adhesion assay with radiolabel	culture medium	
aortic endothelial cells	porcine	60 μM	4.5 h	Propidium Iodide Staining 81.8 ± 5.4%	[17]	fluorescent microscopy with propidium iodide	serum-free culture medium	
neuroblastoma cell line SH-SY5Y	human	60 μM	24 h	cell viability was reduced to 61.68% ± 6.75	[18]	MTT assay	distilled H_2_O and serum-free medium	
zygotes	mouse	30–50 μM	30 min	DNA damage, G2/M cell cycle arrest	[4] (p20)	immunofluorescence	culture medium	
smooth muscle cells	rat	100 μM	4 h	cell cycle arrest in G0/G1	[19]	Northern blot, WB, Fluorescence-activated cell sorting (FACS)	culture medium	
alveolar type II epithelial cells (spontaneously transformed)	mouse	200 μM	5 min	reversible oxidation and inactivation of IKK-β activity	[20]	immunoblotting	culture medium	
osteoblastic OB-6 cells	human	200 μM	48 h	significant cell death if cells weren't pretreated with NrF2 activator (MIND4-17)	[21]	LDH release assay	culture medium	
HeLa cells	human	200 μM	3 h	mitotic arrest	[22]	flow cytometry	culture medium	
SH-SY5Y cell line	human	250 μM	1 h	cell proliferation reduced to ± 15%	[23]	MST assay	not specified	
HeLa cells	human	250–10 000 μM	30 min	the inhibited activity of Aurora A	[24]	WB	culture medium	
hepG2 cells	human	400 μM	24 h	cell viability was reduced to 50.86% ± 1.59	[10]	MTT assay	culture medium	
aortic endothelial cells	porcine	500 μM	90 min	Propidium Iodide Staining 27.6 ± 6.2%	[17]	fluorescent microscopy with propidium iodide	serum-free culture medium	
HEK293	human	500 μM	24 h	cell cycle arrest 24 h after treatment at G2	[25]	flow cytometry	dissolved in PBS, cultured in medium	
mammary adenocarcinoma	mouse	1000 μM	60 min	temporal growth inhibition	[16]	cell count	culture medium	
LHK2 lung adenocarcinoma	human	1000 μM	1 h	higher viability rates than control, acquisition of stemness	[26]	colorimetric assays, SP assay	not specified	
smooth muscle cells	rat	200 μM–1000 μM	24 h	increased apoptosis in a dose-dependent manner	[19]	WB	culture medium	
peripheral blood lymphocytes	human	2000 μM	2–30 min	nuclear transport of STAT3, phosphorylation of STAT3	[7]	WB	culture medium	*inhibited catalase before treatment
her14 (NIH-3T3 fibroblasts)	mouse	3000 μM	0.5–3 h	reversible inhibited cyclin D degradation	[5]	WB	PBSgluc	
her14 (NIH-3T3 fibroblasts)	mouse	5000 μM	1–2 h	no significant lysis	[5]	LDH release	PBSgluc	
oral keratinocytes	human	5000 μM	15 min–8 h	± 85% viable cells	[9]	MTT assay	culture medium	
oral keratinocytes	human	>50 000 μM	8 h	increased necrosis	[9]	annexin-V staining	culture medium	

The generation of ROS is primarily concentrated within specific cellular areas. Due to their short lifespan and heightened reactivity, endogenous ROS inflict the most damage on nearby cellular structures [27]. Main intracellular ROS sources include mitochondria, cytochrome P450, endoplasmic reticulum, and peroxisomes. Within the mitochondria, approximately 1–5% of the oxygen involved in the electron transport chain undergoes reduction, resulting in the formation of $O_{2}^{∙-}$ [28]. Complexes I and III within the respiratory chain are primary sites for the production of superoxide anions [29]. Another major contributor to ROS generation is the family of NADPH oxidase enzymes (NOX), which facilitates the transfer of an electron from cytosolic NADPH to O_2_, resulting in the formation of $O_{2}^{∙-}$ radicals. The impact of ROS on cellular structures depends on the specific ROS involved, their location, concentration, and the cell’s antioxidant defense system. Interestingly, it has been suggested that mitochondrial ROS are involved in cell death, while ROS produced by NOX are associated with promoting cell proliferation and migration [30].

The cell cycle is a precisely regulated process of cell growth and division, influenced by both internal and external signals. Divided into G1, S, G2, and M phases, external signals, like growth factors and internal signals guide the cell through the resting phase (G_0_), differentiation, migration, or proliferation. Each phase depends on a complex network of proteins to ensure proper progression, with checkpoints at G1/S, G2/M, and M monitoring key events.

A key class of regulatory proteins in the cell cycle are cyclin-dependent kinases (CDKs) and their regulators, cyclins. CDKs are serine/threonine protein kinases responsible for phosphorylating substrates to regulate the timing and sequence of cell cycle events. They are activated by binding to specific cyclins at different stages of the cell cycle. CDKs form the catalytic component of heterodimeric kinases, with cyclins acting as the regulatory component responsible for substrate specificity and CDK activity. The accumulation and degradation of cyclins occur cyclically and determine when and where CDKs are activated, making cyclin levels a critical regulatory element of the cell cycle.

CDK activity is regulated by CDK inhibitors (CDKIs), which bind to the ATP-binding site of the CDK complex, inhibiting its ability to phosphorylate target proteins. This inhibition of CDK activity can induce cell cycle arrest, allowing cells to repair DNA damage or enter the G_0_ phase. Two families of CDKIs have been identified based on their structural characteristics: the INK4 proteins and the Cip/Kip family [31]. The INK4 family, comprising p16^INK4a^, p15^INK4b^, and p19^INK4d^, specifically targets CDK4 and CDK6, with no binding to cyclins. On the other hand, the Cip/Kip family, including p21^Cip1^, p27^Kip1^ and p57^Kip2^, inhibits CDK activity by binding to both cyclins and CDKs [31]. Dysregulation of CDKI expression or function is often associated with various diseases, including cancer and neurological disorders. Given the observed overactivity of CDKs in many cancer cells, CDK inhibitors are actively being explored as potential treatments for cancer [32].

The impact of redox changes on the overall state of the cell in individual phases of the cell cycle is well-established, but the potential redox regulatory changes of individual proteins and their subsequent effects on major cellular pathways involved in cell cycle regulation remain unclear and need to be further explored, especially under pathophysiological conditions such as oxidative stress. This review will primarily examine the documented effects of ROS on specific proteins involved in cell cycle regulation. Several academic databases were consulted to complete this review, including PubMed, Google Scholar, Web of Science and Scopus. Combination of keywords such as ‘oxidation’, ‘redox regulation’, ‘reactive cysteines’, ‘oxidative stress’, ‘redox-sensitive proteins’, ‘ROS’, ‘cell cycle’, ‘cell cycle control’ and ‘cell cycle proteins’ were used to search for relevant studies. We excluded studies that showed poor statistical power to reduce the risk of bias and studies that did not report relevant redox-sensitive protein modifications and their effects on cell cycle regulation. Preferred were original experimental studies. Non-peer-reviewed publications were excluded.

### ROS-mediated modulation of protein function

**Figure 1.**
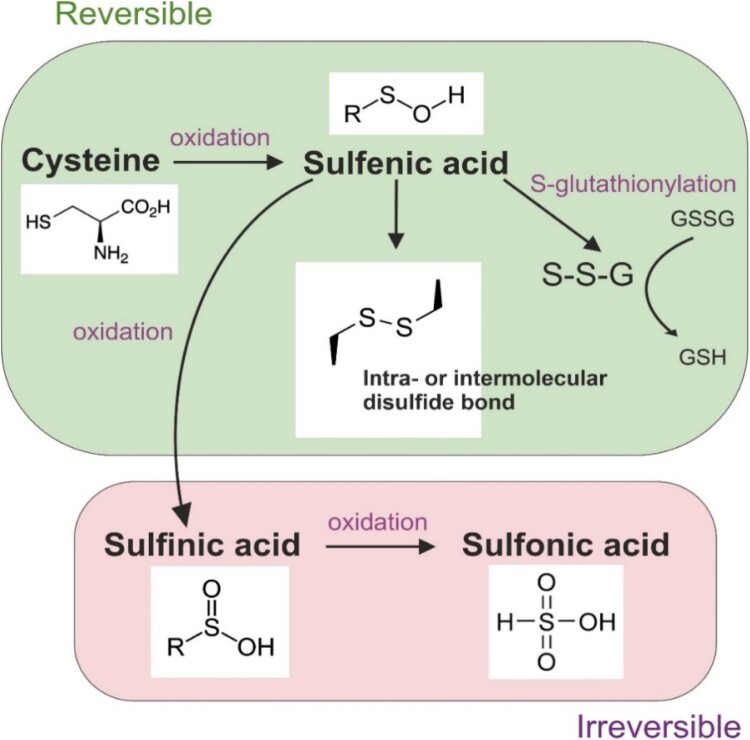
Possible oxidative modifications of redox-sensitive cysteine. The reversible oxidation of thiol groups can result in the formation of sulfenic acid, which can be further oxidized and create intra- or interprotein disulfide bonds or disulfides with glutathione. Irreversible oxidation to sulfinic or sulfonic acid can lead to protein dysfunction and cellular damage.

The oxidation of redox-sensitive cysteine and/or tyrosine residues, particularly in or near the active site of a protein, is a prevalent mechanism of redox signaling. This process critically influences protein structure and function, as the redox state of cysteine residues is a critical factor for proper protein functioning. Protein cysteines can exist as free thiols or participate in structural disulfides within the protein molecule. The reversible oxidation of thiol groups results in the formation of products such as sulfenic acid, which can further react with other thiol groups to form intraprotein or interprotein disulfide bonds or create disulfides with glutathione (GSH) [33] (Figure 1). Irreversible oxidation of cysteine residues to sulfinic or sulfonic acid can lead to loss of protein function and damage cellular processes [33]. The reversibility of these oxidative modifications, alongside the potential for irreversible thiol oxidations, serves as an important cellular mechanism for assessing and addressing the extent of oxidative stress-induced damage [33,34]. Irreversible thiol oxidations can signal high oxidative damage and subsequently trigger cell death [34].

Reactive cysteine residues within antioxidant proteins undergo specific and regulated redox changes in their catalytic cysteines [29]. This mechanism plays a role not only in reducing oxidized target proteins, protecting them from further oxidative damage, but also acts as a detector of harmful oxidative levels within cells [29]. Moreover, the oxidation of cysteine residues may potentially displace specific metal cofactors, such as zinc ions within zinc finger motifs, disrupting the function of proteins featuring this motif, including certain transcription factors, kinases, and phosphatases where zinc or iron serves as a critical cofactor [3,34–37]. In addition to cysteine, protein tyrosine residues can undergo oxidation, resulting in the formation of tyrosyl radicals that initiate free radical chain reactions [38]. The reversible oxidation of tyrosine residues to form sulfides can serve as a redox switch, regulating protein activity and signaling pathways.

The sensitivity of cysteine residues to redox changes is influenced by their steric accessibility and the pKa value of their thiol groups. Typically, the pKa value of free cysteine is approximately 8.2 [39]. However, when cysteine residues are located near positively charged residues, their pKa drops to less than 6.5, making them more susceptible to oxidation [34,39]. This shift in pKa is associated with transforming redox-sensitive thiols into potent nucleophiles in the presence of basic residues [29,40]. Additionally, the local pH can influence the pKa value of cysteine residues [41].

### Antioxidant defense

The balance between ROS production and antioxidant defense is vital for maintaining cellular homeostasis. When the cellular redox system is overwhelmed with oxidized proteins and lacks sufficient reduced counterparts for their reduction, oxidative stress arises. In cases of temporary stress, proteins may undergo reversible oxidation, but in instances of substantial or persistent imbalance, irreversible oxidative changes can take place. Therefore, cells have developed a sophisticated antioxidant defense system to prevent excessive oxidative damage and maintain favorable redox homeostasis.

Detoxification of ROS is achieved through a complex interplay of enzymatic and non-enzymatic molecules, including GSH, NADPH, thioredoxin (Trx), superoxide dismutase (SOD), glutathione peroxidase (GPx), glutathione reductase (GR), peroxiredoxin, and catalase (CAT). Superoxide radicals are converted into H_2_O_2_ by various isoforms of SOD (Cu/Zn-SOD, Mn-SOD, extracellular SOD), with catalase further converting H_2_O_2_ into water and oxygen [42]. SOD isoforms are compartmentalised within specific cellular regions; for instance, cytoplasmic Cu/Zn-SOD is the main contributor to overall SOD activities, while Mn-SOD is the main mitochondrial antioxidant enzyme [43]. Notably, elevated Cu/Zn-SOD expression has been associated with increased proliferation and invasiveness in tumor cells [44]. Inhibition of Cu/Zn-SOD has been shown to induce cell cycle arrest in G1 and apoptosis [43]. Cu/Zn-SOD overexpression has been linked to the positive regulation of Carnitine Palmitoyltransferase 1A (CPT1A), which may explain its role in promoting cancer cell growth [44]. CPT1A is involved in cellular lipid metabolism, facilitating the entry of fatty acids into mitochondria and contributing to increased energy production.

Heme Oxygenase-1 (HO-1) is an enzyme that degrades heme, producing biliverdin, carbon monoxide (CO), and free iron. Biliverdin and CO contribute to the antioxidant effects of HO-1, protecting cells against oxidative stress and inflammation. However, elevated levels of HO-1 can have a pro-oxidant effect due to the release of free iron and consequent Fenton reaction [45]. Intriguingly, HO-1 also promotes ferritin expression, essential for sequestering and storing free iron, mitigating its potential pro-oxidant effects. The role of HO-1 in cancer cell proliferation is gaining attention, with implications in various pathways such as BCR/ABL, c-Met–Ras, and EGFR–Src–NF-κB signaling to promote cell proliferation [45–48].

GPx plays an important role in targeting and reducing lipid hydroperoxides and H_2_O_2_, safeguarding cellular membranes. The catalytic mechanism of GPx involves the use of GSH, and the resulting transformed form, glutathione disulfide (GSSG), is enzymatically converted back to its original state by glutathione reductase [42]. Thioredoxin, a redox-active protein, contributes to antioxidant defense by reducing disulfide bonds in oxidized proteins. It has a role in cellular signaling and gene expression regulation, and the reduction of its oxidized form is catalyzed by thioredoxin reductase (TR), utilizing NADPH as an essential cofactor in the redox reaction [35]. Additionally, peroxiredoxins often utilize thioredoxin as an electron donor during the reduction of peroxides [49].

In response to oxidative stress, cells undergo distinct metabolic adaptations, altering their energy production and utilization. This includes a preference for glycolysis even when oxygen is available, known as the Warburg effect. Activating the pentose phosphate pathway (PPP) is crucial as it generates NADPH for antioxidant defenses and facilitates the recycling of key antioxidants, such as glutathione and thioredoxin. This metabolic shift also supports lipid production, essential for repairing and maintaining cell membranes [50].

The antioxidant response can be indirectly triggered by oxidative stress. Inhibitors of the antioxidant response's effectors often contain a reactive cysteine residue, leading to the dissociation of inhibitor-target complexes upon cysteine oxidation [34]. For example, the NF-E2-related factor 2 (Nrf2) family of transcription factors serves as the first-line defense activated by low increases in ROS levels [51]. Nrf2 belongs to the cap'n'collar family of basic leucine zipper transcription factors. Under normal conditions, Nrf2 remains inactive in the cytoplasm, repressed by Kelch-like ECH-associated protein 1 (Keap1) [52]. During oxidative stress, KEAP-1 forms a disulfide dimer at Cys151, losing its ability to bind Nrf2 [53]. Activated Nrf2 then orchestrates the upregulation of antioxidant genes like SOD1, CAT, GPx, Trx, and HO-1, enhancing cellular resilience [45,54].

Another example of redox-regulated transcription factor is nuclear factor-κB (NF-κB). NF-κB is a family of transcription factors including p50, p52, p65, RelB, and c-Rel, regulated by inhibitory proteins (IκBs) in the cytoplasm [55]. Upon activation triggered by stimuli like cytokines, pathogens, or stress, IκB undergoes phosphorylation by IKK (IκB kinase) and degradation in the proteasome, releasing NF-κB. Interestingly, IKK can be directly S-glutathionylated by H_2_O_2_, inhibiting its function [20]. Similarly, the oxidation of native p50 leads to its glutathionylation, partially inhibiting its DNA binding ability [56]. Once freed, NF-κB translocates to the nucleus, regulating gene transcription in response to changes in cell homeostasis. NF-κB is directly influenced by the TRX system, whereas it does not respond directly to the GSH system [57]. In the cytoplasm, increased TRX levels inhibit the degradation of IκBα [57]. Within the nucleus, TRX directly reduces cysteine modifications on NF-κB, enabling NF-κB-dependent gene expression. NF-κB plays a dual role in cellular redox balance, protecting against ROS by inducing the expression of antioxidant proteins like MnSOD, HO-1, or thioredoxin, while also assuming a pro-oxidant role by promoting the expression of genes such as the NOX2 subunit of NADPH oxidase [55,58,59]. Prolonged oxidative stress has been observed to inhibit NF-κB activation by inactivating the proteasome and impeding I-κB degradation [13,55]. Furthermore, the complex interplay between Nrf2 and NF-κB shapes cellular responses to oxidative stress, with Nrf2 knockout enhancing NF-κB activity [60]. Induced by Nrf2, HO-1 inhibits NF-κB [61], while NF-κB suppresses Nrf2 by competing for the CBP–p300 complex [62].

Metallothionein (MT) is a family of small, cysteine-rich proteins that bind heavy metal ions like zinc, copper, and cadmium. This family plays a key role in maintaining cellular metal homeostasis, detoxification, and shielding against metal toxicity, contributing to the cellular response to oxidative stress [63]. A high GSH/GSSG ratio inhibits the release of zinc from the MT molecule [64]. The zinc stored within MT molecules remains unavailable for the activation of specific target proteins that rely on zinc for their active conformation. This includes transcription factors with a zinc motif or p53, which utilizes zinc to stabilize its binding to DNA [65–67]. Moreover, MT engages in antiapoptotic mechanisms by interacting with the p50 subunit of NF-κB, leading to the transactivation of the antiapoptotic NF-κB pathway [67]. During oxidative stress, the depletion of free GSH coincides with the oxidation of thiol groups in cysteines within the MT molecule, resulting in the release of free zinc (II) [63]. Released zinc (II) subsequently regulates various target proteins, initiating a positive feedback loop where it supports the expression of MT and activates Nrf2 [68]. Nrf2, in turn, triggers the transcription of antioxidant enzymes such as HO-1 and CAT [68]. MT concentration peaks in the G1 and G1/S phases of the cell cycle [69]. In tumor cells, MT typically plays a protective role and contributes to cell proliferation [70], although exceptions exist [67]. For instance, phosphorylation induced by MT2A overexpression may inhibit cell proliferation through the inhibition of the Hippo signaling pathway [63].

## Redox status of the cell

Redox potential serves as a measurable indicator, reflecting a system's readiness for redox reactions by indicating its ability to donate or accept electrons. This property significantly influences the dynamic equilibrium of redox couples including NADH/NAD+, NADPH/NADP+, and GSH/GSSG. The GSH/GSSG ratio serves as a valuable indicator, often used to assess the cell's overall redox condition [35]. GSH concentration is evenly distributed in the cytoplasm and nucleus, it peaks during the S and G2/M phases, and its depletion leads to reduced proliferation and apoptosis [35].

The intracellular redox potential typically ranges from −160 to −260 mV, with cell death often occurring when it rises above −160 mV [71,72]. In noncancerous cells, a redox potential of approximately −207 mV has been identified as a threshold significant for pRB phosphorylation above which the cell cycle stops [71,72]. Intervening with antioxidants during the G1 phase induces a shift towards a more reducing redox state, resulting in increased hypophosphorylated pRB and subsequent cell cycle arrest [73]. As cells progress to the S phase, their redox potential decreases, falling below the threshold for pRB dephosphorylation [72]. Notably, the impact of oxidative stress on cells is closely tied to specific phases within the cell cycle; fibroblasts exposed to H_2_O_2_ undergo apoptosis in the S phase, while in G1 or G2/M phases, they stop proliferating [11]. The cell's redox state emerges as a promising modulator of cell cycle progression, and antioxidant intervention holds the potential to regulate cell proliferation under pathophysiological conditions. However, pinning a definitive value to the cell's instantaneous overall redox potential remains challenging due to varying redox potentials among different cellular components [74].

## ROS impact on cell proliferation and progression through G1 phase

In the G1 phase of the cell cycle, cells decide whether to continue dividing or enter a state of rest known as quiescence or the G_0_ phase. In G_0_, cells focus on essential activities, and to re-enter the cell cycle, external cues like growth factors, hormones, and cytokines are needed.

**Figure 2.**
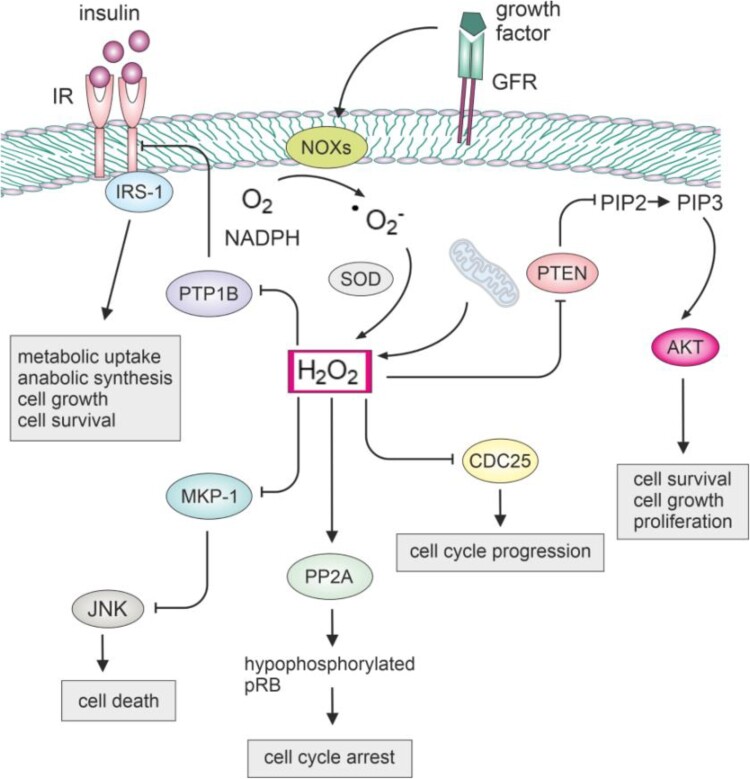
Impact of hydrogen peroxide on the function of key cellular phosphatases. NOX produces superoxide, which can be converted to H_2_O_2_ by SOD. The generated H_2_O_2_ can then modulate redox-sensitive phosphatases. Inhibiting PTP1B may enhance insulin signaling, potentially improving glucose homeostasis. This can positively impact cell growth and metabolism. Inhibiting MKP-1 may lead to prolonged JNK activation, affecting stress signaling, apoptosis, or inflammatory processes. Inhibiting CDC25 could disrupt cell cycle progression, inducing cell cycle arrest. Inhibiting PTEN may lead to increased Akt signaling, promoting cell survival and growth. Dephosphorylation of pRB by PP2A results in the inhibition of E2F transcription factors, preventing the progression of the cell cycle from the G1 to the S phase.

Ligand binding to growth factor receptors initiates a complex network of downstream signaling pathways. This process can trigger the production of H_2_O_2_ in nanomolar concentrations through the activity of NADPH oxidases like Nox1 and Nox4 [75,76]. Generated H_2_O_2_ can play a critical role in oxidizing and inactivating key phosphatases, that normally act to suppress proliferative pathways, as well as protein kinases. Specifically, redox-sensitive phosphatases, including protein tyrosine phosphatase 1B (PTP1B), phosphatase and tensin homolog (PTEN), protein phosphatase 2A (PP2A), Cdc25, and MAP kinase phosphatases (MKPs), are susceptible to oxidation due to the presence of cysteine with a low pKa in their catalytic sites (Figure 2) [77–80]. Their inactivation can result in the physiological activation and amplification of proliferation signaling, but it may also contribute to the dysregulation of cell signaling and promote unchecked proliferative pathways. By oxidizing and inactivating JNK phosphatases within the MKP family in NF-κB deficient cells, H_2_O_2_ induced prolonged activation of JNK, leading to cell death [14]. Oxidation of PTEN induces the formation of a disulfide bond, resulting in the inactivation of its phosphatase activity [81]. Consequently, PTEN loses its ability to dephosphorylate PIP3, leading to the sustained activation of PI3 K/Akt signaling [15,82].

**Figure 3.**
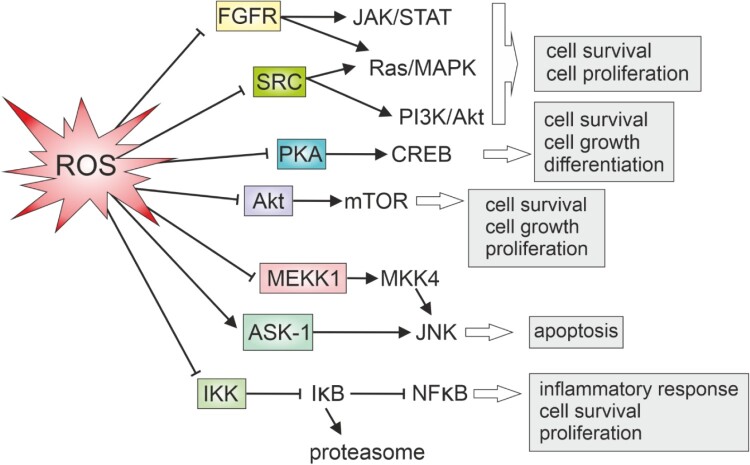
Oxidative regulation of key protein kinases and cellular outcome.

Protein kinases, including members of the Src and fibroblast growth factor receptor (FGFR) families, are no exception from the adverse effect of oxidants (Figure 3). Specifically, protein tyrosine kinases (PTKs) within these families can be directly affected by oxidation [83,84]. In the case of the Src family, Cys277 in the catalytic domain undergoes oxidation, resulting in the homodimerization of Src molecules connected by a disulfide bridge [84]. This selective oxidative inactivation is prominent in crucial members of the Src family and all kinases within the FGFR family [84]. Similarly, cAMP-dependent protein kinase (PKA) is also explored as a target for oxidative regulation, since it is susceptible to reversible inactivation by glutathionylation [83].

Protein kinase B, also known as Akt, is activated during growth factor signaling through sequential phosphorylation at Ser473 by mTORC2 and Thr308 by 3-phosphoinositide-dependent kinase 1 (PDK1) [85]. Low levels of H_2_O_2_ promote Akt activation, possibly by oxidizing Cys310 to sulfenic acid near the catalytic loop. This oxidation may enhance Akt binding to PDK1, favoring Thr308 phosphorylation. Conversely, moderate to high H_2_O_2_ concentrations induce the oxidation of Akt1 Cys60 in the PH domain and Cys310 to sulfonic acid [85]. In this oxidative environment, Akt and PDK1 binding is disrupted, preventing the second phosphorylation of Akt at Thr308 [85]. Consequently, monophosphorylated Akt accumulates in mitochondria, leading to increased GSK-3α/β activity, reduced pro-survival signaling, and cytochrome c release, ultimately inducing apoptosis [85].

MAPKs, including extracellular signal-regulated kinases (ERKs), c-Jun N-terminal kinases (JNKs), and p38 MAPK, contain cysteine residues sensitive to oxidation [86–88]. The oxidation of these cysteine residues can induce conformational changes, influencing both kinase activity and downstream signaling.

According to one established model, Ask-1, a member of the MAP3K superfamily, interacts with reduced thioredoxin which acts as its inhibitor [89]. Upon exposure to oxidants, thioredoxin undergoes oxidation, leading to its dissociation from the Ask-1 complex. Subsequently, Ask-1 can oligomerize, initiating downstream signaling for apoptosis [89]. However, a more recent perspective suggests that H_2_O_2_ treatment oxidizes Ask1 directly and induces the formation of disulfide bonds between Ask1 molecules [87]. Both phosphorylation and oxidation of Ask1, occurring independently, are considered crucial for competent signaling to MKK4/7, JNK, and apoptosis. While phosphorylation alone is adequate for Ask1 activation, oxidation plays a significant role in the subsequent phosphorylation of MKK4/7 and JNK. Notably, Trx1 transiently associates with Ask1 in this model, exerting a negative regulatory role by reducing Ask1 multimers [87].

Similarly, within the MEKK1 (Mitogen-Activated Protein Kinase Kinase 1) domain, glutathionylation of cysteine residues induced by elevated oxidant levels results in the dysfunction of its kinase activity [90]. MEKK1 is linked to stress responses and inflammation signaling, impacting downstream proteins like p38 MAPK or JNK in stress response signaling.

Furthermore, H_2_O_2_ is essential in autophagy by oxidizing cysteine residues in the structure of Atg4, a cysteine protease involved in autophagy. The oxidation inhibits the function of Atg4 and facilitates the required lipidation of Atg8, contributing to the autophagic process [91]. Autophagy can be linked with cell cycle arrest and senescence, as reviewed elsewhere [92]. The interplay between autophagy and cell cycle progression is evident from various signaling pathways, such as the involvement of AMBRA1 in both autophagy promotion and inhibition of cell proliferation via c-Myc regulation [92]. Furthermore, CDKIs like p16, p21, and p27 are known to induce autophagy, suggesting coordinated stress responses connecting autophagy induction and cell cycle arrest [92]. Additionally, autophagy proteins like Atg5 and Atg7 contribute to establishing senescence [92].

The cyclin D-CDK4/6 complex has a main role in directing the cell cycle progression through the G1 phase, with its activity finely tuned by signaling events triggered by external cues for proliferation and growth. It was recently found that the oxidation of CDK4 triggers the formation of a reversible intermolecular disulfide bond between Cys135 of CDK4 and Cys7/8 of cyclin D, which inhibits cyclin D-CDK4 activity, consequently reducing cell proliferation in a mouse model of pulmonary hypertension [93]. The activation of cyclin D promoters involves the integration of various pathways, including Ras-Raf-MEK-ERK, PI3K-Akt, and Wnt signaling [94]. These pathways converge to activate specific transcription factors, such as AP-1, STAT, TCF, and c-myc, which bind to the consensus sequence within the cyclin D promoter and initiate its transcription [94]. For example, in cardiac cell proliferation, Nox4-generated ROS triggers ERK1/2 activation, leading to c-myc phosphorylation and an increase in cyclin D2 expression [76].

The AP-1 (Activator Protein 1) transcription factor plays a crucial role in initiating cyclin D transcription. It consists of various Jun family proteins (c-Jun, JunB, JunD) and Fos family proteins (c-Fos, FosB, Fra-1, Fra-2) [95]. Specifically, c-Jun, Fra-1, and Fra-2 act as activators in cyclin D1 transcription. In contrast, c-Fos has a dual role, functioning as both a depressor and activator of the cyclin D1 promoter at different stages [96]. Meanwhile, JunB consistently acts as a repressor in regulating the cyclin D1 promoter [96]. Upon growth factor stimulation in quiescent cells, c-Fos is synthesized and translocated to the nucleus. Subsequently, its ERK-mediated phosphorylation and degradation occur, facilitating Fra1 protein access to chromatin and initiation of cyclin D1 expression [97]. However, heightened H_2_O_2_ levels cause hyperactivation of ERK1/2, hindering cell cycle progression by stabilizing c-Fos binding to DNA and impeding Fra-1 access to chromatin, resulting in blocked cyclin D1 expression [97,98]. In contrast, Munõz [5] observed that millimolar concentrations of H_2_O_2_ maintained cyclin D levels at a consistent level in Her14 fibroblasts. This discrepancy in findings might be attributed to the timing of hydrogen peroxide application, potentially occurring after cyclin D was already expressed within the cell. Notably, the observation that inhibiting cellular protein synthesis had no impact on cyclin D levels further supports this possibility. The other potential mechanism underlying the observed effect on cyclin D expression is that H_2_O_2_ interferes with the ubiquitin-proteasome degradation of cyclin D [5], which is further supported by another study where sustained oxidative stress led to proteasome inhibition [13].

Additionally, Menon et al. [99] conducted a study involving the treatment of mouse fibroblasts with antioxidant N-acetylcysteine (NAC), resulting in an increased proportion of cells arrested in the G1 phase accompanied by a notable decrease in cyclin D1 levels. This effect was coupled with later elevation in MnSOD levels. Interestingly, after NAC application, the level of superoxide rose, particularly in cells with lower MnSOD levels, leading to a more pronounced G1 arrest. MnSOD's role in converting superoxide to H_2_O_2_ supports the possibility of a rapid rise in superoxide radicals after treatment with NAC. These findings collectively suggest that NAC may have an immediate prooxidant effect, contrasting its longer-term antioxidant capabilities. Notably, the expression of D cyclins is transient, with rapid degradation upon withdrawal of mitogenic signals. Cyclin D possesses two phosphorylation sites at T286 and T288, and withdrawal of growth factors activates glycogen synthase kinase-3 Beta (GSK-3Beta), marking D cyclins for ubiquitination and eventual degradation. The study suggests redox-sensitive regulation of T286 phosphorylation in cyclin D degradation, as nondegradable T286A mutants’ levels of cyclin D didn't change after NAC treatment [99].

The signal transducer and activator of transcription 3 (Stat3) is known to play a role in promoting cell cycle progression, particularly from the G1 phase to the S phase. It regulates key proteins like cyclin D and CDKs. Stat3 possesses redox-sensitive cysteines, and their inactivation through mutation resulted in increased cell proliferation [100]. Genes linked to immune function and cell adhesion were observed to be indirectly upregulated via Stat3 in response to oxidative stress, while genes displaying reduced activity were associated with developmental processes and cell death [101]. Additionally, a synergistic interplay between Stat3 and Hif-1α in response to oxidative stress was described [101]. In a study conducted by Xie et al. [102], mild oxidizing conditions induced S-glutathionylation of Stat3 in HepG2 hepatocarcinoma cells. This post-translational modification adversely affected Stat3's functional state, influencing nuclear translocation, phosphorylation status, and, consequently, its ability to execute transcriptional functions. Furthermore, the altered Stat3 exhibited reduced favorability as a substrate for other signaling proteins, including JAK2 [102]. In contrast, a different study demonstrated that H_2_O_2_, in conjunction with vanadate, induced tyrosine phosphorylation of Stat3, leading to its subsequent translocation into the nucleus in peripheral human lymphocytes [7]. The authors hypothesized that the stimulating effects of oxidants, specifically H_2_O_2_, might arise from the inhibition of intracellular PTPases. They also suggested that intracellular Fe^2+^/Cu^2+^ ions could potentially serve as intermediates under conditions of H_2_O_2_-induced oxidative stress, enhancing the nuclear translocation of Stat3 [7].

The contrasting effects of oxidants, not only on Stat3 function, observed in various studies may be attributed to variations in experimental design. Notably, different cell lines were employed, potentially yielding distinct responses to similar stimuli. The choice between cancerous and normal cell lines further introduces variability in the redox environment and cellular responses. Additionally, variations in the treatments, encompassing differences in chemical agents, duration, and other conditions, could contribute to the divergent outcomes. This heterogeneity in experimental parameters emphasizes the importance of considering diverse factors when interpreting the redox regulation of proteins and its implications for cell cycle dynamics.

The cyclin D-CDK4/6 complex phosphorylates the tumor suppressor pRB (retinoblastoma), a key regulatory event that tightly governs the progression of the cell cycle. The RB protein is hypophosphorylated upon entry into the G1 phase of the cell cycle and loses its inhibitory function. Monophosphorylation of the RB protein by the cyclin D-CDK4/6 complexes creates a transition between entry into the G_0_ phase and commitment to continue the cell cycle. The hyperphosphorylated RB protein is inactivated and releases E2F transcription factors, which can activate transcription in the nucleus, leading the cell to enter the next phase of the cycle. E2F-activated genes are responsible for cell cycle progression, DNA replication, genome protection, and cell growth. Transcription of E cyclins by E2F causes activation of CDK2, and this complex further phosphorylates pRB, leading to its complete inactivation and transition from G1 to S phase [3]. A study by Cicchillitti et al. [103] investigated the impact of H_2_O_2_ on retinoblastoma family proteins (pRb, p107, and p130) in endothelial cells. H_2_O_2_ induced rapid hypophosphorylation of these proteins, mediated by PP2A. Inhibiting PP2A prevented hypophosphorylation, and its activity positively correlated with H_2_O_2_ treatment [103]. Moreover, PP2A inhibition prevented H_2_O_2_-induced DNA synthesis inhibition, suggesting a role for PP2A-mediated dephosphorylation of pRB in the cellular response to oxidative stress [103].

## DNA damage and S phase

During the S phase, also known as the synthesis phase, the cell undergoes genetic material duplication in preparation for subsequent cell division. This phase involves critical processes, including the proofreading and repair of DNA errors to ensure accurate genetic duplication.

CDK2 plays an important role during the transition from the G1 to the S phase. It transitions from binding cyclin E to cyclin A. During the transition phase, the negative regulator Kinase Associated Phosphatase (KAP) gains access to the T-loop of CDK2. This interaction between CDK2 and KAP is tightly regulated by the oxidation of CDK2 to sulfenic acid on Cys177, which is physiologically caused by mitochondrial ROS. This oxidation ensures the phosphorylation of CDK2 in the T-loop, a modification that obstructs KAP binding and facilitates the subsequent activation of CDK2 [104]. The study by Deshpande et al. [19] demonstrated that H_2_O_2_ had a dual impact on cell cycle regulators. Surprisingly, H_2_O_2_ initially increased CDK4 activity, potentially propelling cells from quiescence to mid-late G1. However, this was followed by a subsequent inhibition of cyclin A–CDK2 activity, causing cell cycle arrest at the G1/S interface. In response to serum, H_2_O_2_ completely suppressed cyclin A mRNA, essential for G1/S and S phase progression, while cyclin D1 mRNA remained unaffected [19]. Additionally, H_2_O_2_ induced a significant increase in the expression of the cell cycle inhibitor p21, with no observed change in p27 protein levels. The heightened levels of p21 protein highlight its potential role as a major effector in H_2_O_2_-induced growth arrest [19].

A recent study conducted by Kirova et al. [104] has presented compelling evidence that showcases the essential role of oxidants in managing cell cycle progression during the S-phase. The study findings reveal that antioxidant treatment leads to a dose-dependent reduction in proliferation. The study effectively reduced mitochondrial ROS production by limiting the metabolite supply to the TCA cycle in mitochondria. Consequently, this alteration led to a slower proliferation rate and an extended duration of the S phase. The reduced formation of mitochondrial ROS was also associated with a delayed onset of DNA replication in the S phase, suggesting a direct involvement of ROS in promoting DNA replication [104]. However, this observation may be attributable to the disruption of mitochondrial metabolism, whereby metabolites essential for DNA synthesis might become unavailable. The observed natural increase in ROS levels during the S and G2/M phases could be a consequence of heightened mitochondrial activity, including the shift from glycolysis to oxidative phosphorylation and an increase in mitochondrial mass [104].

Before DNA replication, the cell evaluates nutrient, energy, and growth factor availability, and checks for DNA damage. The Intra-S phase checkpoint maintains replication fork integrity, ensuring accurate DNA replication, and enables a seamless transition into the mitotic phase. Activated in response to various forms of damage, including oxidative stress, this checkpoint suppresses the activation of late replication origins [105]. Oxidative stress-induced DNA damage manifests in a variety of deleterious effects on genetic material, such as the conversion of guanine to 8-oxoguanine (8-oxoG), alterations in base pairs, the formation of apurinic/apyrimidinic (AP) sites, single-strand breaks (SSBs), double-strand breaks (DSBs), mutations, changes in gene expression and increased susceptibility to cancer [88]. The MRN (MRE11-RAD50-NBS1) complex recognizes DNA damage and activates the damage sensor and signal transducer protein kinase ataxia-telangiectasia mutated (ATM), which, in turn, phosphorylates numerous substrates. Among these substrates, Chk1/2 and p53 are considered the most crucial players in the cellular response to DNA damage. Elevated levels of ROS activate ATM, which in turn can lead to cell cycle arrest through the ATM-Chk2 pathway [106]. Inhibiting ATM under elevated levels of oxidants did not activate p53 and Chk2, indicating that oxidative stress primarily activates p53 through ATM [107].

In DNA repair, apurinic/apyrimidinic endonuclease-1 (APE1) is needed for the base excision repair (BER) pathway, addressing damage to single-stranded DNA (ssDNA) and AP sites from ROS or alkylating agents [108,109]. The protein's expression peaks during the S phase of the cell cycle, signifying an increased risk of DNA damage due to heightened DNA replication activity and ROS production [108]. H_2_O_2_-induced APE1 expression enhances overall cellular resistance to oxidative stress [108]. Moreover, APE1 enhances AP-1 binding to DNA by reducing the conserved Cys272 residue in the DNA-binding domain of c-Jun, and the oxidation of APE1 significantly impairs this ability which can lead to a decrease in cyclin D expression [108,110,111]. APE1 possesses seven conserved cysteines implicated in redox regulation, but their accessibility varies. While some studies point to Cys65 as an active redox site, Bhakat's protein model suggests its limited accessibility for oxidation [108,112–114]. Conversely, Howpay Manage et al. [109] detected oxidation of five out of seven cysteines in APE1, including Cys65, when exposed to CO_3_•– [109].

P53, a critical tumor suppressor, plays a key role in the cellular response to DNA damage, capable of inducing cell cycle arrest, DNA repair, or apoptosis upon activation [115]. The multifaceted and context-dependent nature of oxidation in p53 function is a subject of ongoing research. In response to oxidative stress, p53 activates the transcription of genes such as p21 and redox enzymes like Gpx1 and MnSOD, forming a protective mechanism against the damaging effects of ROS [71,88,116,117]. Accordingly, reduced p53 activity contributes to ROS accumulation, further supporting its antioxidant function [71,117,118]. However, some studies propose a potential pro-oxidant role for p53, linking its activation to increased ROS levels and apoptosis induction, while also functioning as a transactivator for prooxidative genes like PIG3 and PIG6 [88,119,120]. It was shown that p53 counteracts Nrf2-induced transcription of certain antioxidant genes, suggesting a negative control mechanism to prevent the generation of a robust antioxidant environment that might impede the induction of apoptosis [120].

The dual nature of p53's effects on ROS and apoptosis depends on cellular levels of oxidants and their signaling duration. Transient oxidation tends to induce temporal cell cycle arrest and antioxidant function of p53, while prolonged induction or high oxidative stress are associated with apoptosis and pro-oxidative effects of p53 signaling [71,120,121]. Oxidation of specific cysteine residues in p53's DNA-binding domain can impact the protein's ability to bind to specific DNA sequences. P53 has ten cysteine residues in the DNA-binding domain [88]. Cys182 has been identified as sensitive to oxidation, potentially leading to structural damage in the p53 protein [122]. On the other hand, oxidation of Cys277 may contribute to the recognition of specific p53 response elements in genes, enhancing their activation in response to redox regulation [88,123]. In conclusion, the role of oxidation in p53 function is complex and dynamic, involving a delicate balance between pro-oxidant and antioxidant effects. The context-dependent nature of p53's response to oxidative stress underscores the need for further research to identify the molecular mechanisms underlying its dual role in maintaining cellular redox homeostasis.

## G2, M phase, and ROS influence

In the G2 phase of the cell cycle, DNA replication is finalized, and the cell readies itself for mitosis. This phase also acts as a checkpoint to detect any DNA damage or incomplete replication that could potentially cause problems. The CDK2/cyclin A complex is involved in the control of DNA replication during the S phase and is still active in the G2 phase, contributing to the regulation of the G2/M transition [124]. CDK1 (or CDC2) and cyclin B complex have a main role in the progression from G2 to mitosis – the levels of cyclin B rise during the G2 phase and peak at the G2/M transition. As A/B-CDK1 activity surpasses a threshold, the prophase of mitosis is initiated. This elevation in A/B-CDK1 activity leads to the phosphorylation of numerous CDK1 substrates, including mitotic kinases. It induces cellular rounding, chromatin condensation within the nucleus, nuclear envelope disassembly, and permeabilization [32].

Cdc25 (Cell Division Cycle 25) phosphatase enzymes belong to PTPs and play a key role in cell cycle progression by activating CDKs through dephosphorylation. Specifically, Cdc25B and Cdc25C are crucial in regulating G2/M transitions through dephosphorylating Cdk1 [125,126]. The activation of Cdc25C in the G2/M transition involves its dissociation from 14-3-3 proteins [126] (p201). The activation of Cdc25B in the G2/M transition involves its phosphorylation by PLK1, PKA, and Aurora-A kinase [126]. The phosphorylated form of Cdc25B concentrates at the centrosome, where it colocalizes with Aurora-A [126,127].

In response to H_2_O_2_ exposure, disulfide bonds form between Cys377 and Cys330 in the Ccd25C, leading to the inactivation of its function [128,129]. The active-site cysteine in Cdc25 can be shielded from rapid oxidation to irreversibly inactivated sulfinic acid by swiftly forming intramolecular disulfide bonds with adjacent (back-door) cysteines. This protective mechanism prevents the potential consequences of irreversible oxidation and is efficiently reduced by thioredoxin/thioredoxin reductase [129].

The Anaphase-Promoting Complex/Cyclosome (APC/C) is a multi-subunit E3 ubiquitin ligase with the primary function of targeting specific proteins for ubiquitination and subsequent degradation by the proteasome. Cdh1, or Cdc20 Homolog 1, is an activator subunit of APC/C, particularly in late mitosis and the G1 phase. Its activation of APC/C allows it to recognize and ubiquitinate its substrates such as cyclin A, cyclin B, and securin, promoting the progression to anaphase, while securin degradation releases separase, which cleaves cohesin and separates the chromatids. It also initiates the formation of an actomyosin contractile ring that contracts during cytokinesis, dividing the cell into two. Cdh1 is phosphorylated by CDK1, leading to inhibition of its interaction with APC/C. With an increased level of endogenous H_2_O_2_, coordination cysteine is oxidized in the APC11 unit of the APC complex, causing the display of zinc from the molecule resulting in APC's inability to bind to the enzyme ubiquitin-conjugating enzyme E2 4 (Ubc4) [6,130]. Ubc4, as an E2 ubiquitin-conjugating enzyme, catalyzes the transfer of ubiquitin molecules to target proteins, marking them for degradation by the proteasome.

In prometaphase, the nuclear envelope disintegrates, allowing condensed chromosomes to interact with microtubules, thereby prompting the formation of the mitotic spindle [32]. Kinases such as Plk, Aurora A, and Aurora B actively contribute to spindle assembly and connection. The activity of Aurora kinases is triggered by auto-phosphorylation at conserved threonine residues, mediated by co-factors [131]. It was found that oxidative stress caused hyperphosphorylation of Aurora A at the beginning of mitosis, abnormal mitotic spindle formation, stabilized levels of cyclin B, and a significantly delayed mitosis [22]. Moreover, other studies pointed out that increased phosphorylation of Aurora A at T288 by PP1[132], PAK1[133], or PP6[134] can negatively affect its function [22]. It was also discovered that the catalytic activity of the Ser/Thr kinase Aurora A is inhibited when the conserved cysteine residue (Cys290) adjacent to the critical phosphorylation site Thr288 undergoes oxidation [24]. Similarly, a study by Chang et al. [6] observed that H_2_O_2_ inhibits cyclin B degradation and delays mitosis; however, these results had been attributed to the oxidation of APC/C [6].

Contrary to this, Lim et al. [135] proposed a different perspective. They found that during the cell cycle, the centrosome is shielded from endogenous H_2_O_2_ influence by the H_2_O_2_-eliminating enzyme PrxI. At the beginning of mitosis, PrxI is inactivated by Cdk1, causing a temporary increase in H_2_O_2_ levels in the centrosome. This increase deactivates phosphatases that negatively regulate Cdk1, especially Cdc14B, near the centrosome, leading to further activation of Cdk1 [135]. The experiment demonstrated that H_2_O_2_ is necessary during mitosis to enhance the full activation of Cdk1 [135]. This finding could potentially explain why persistent H_2_O_2_ signaling results in increased hyperphosphorylation of Aurora A and halted degradation of cyclin B, with the cause potentially being sustained signaling of active Cdk1. It is noteworthy that full activation of Aurora during G2/M is downstream of Cdk1 [136], and activated Cdk1 inhibits APC/C. This inhibition ensures the prevention of cyclin B degradation. Taken together, this suggests that the temporal rise of endogenous H_2_O_2_, produced under physiological conditions, may be crucial for mitotic transition, while unnatural levels and timing of its activity could lead to mitotic arrest, potentially caused by sustained CDK1 signaling. However, the specific mechanisms of this outcome and the role of prolonged CDK1 activation require further exploration.

## Therapeutic strategies

ROS are critical regulators of the cell cycle, exerting both beneficial and detrimental effects depending on their concentration, duration of action, and the cellular context. At physiological levels, ROS act as signaling molecules that promote normal cell cycle progression and maintain cellular homeostasis. ROS regulate key physiological processes, including cell proliferation, differentiation, and survival through the activation of signaling pathways such as MAPK, PI3 K/Akt, and NF-κB, which are critical for cell cycle progression, immune response, and homeostasis. ROS also modulate the activity of cyclins and CDKs, which are key regulators of the cell cycle. In 2003, Nick Lane introduced a theory of ageing and disease termed the double-agent theory [137]. Central to this theory is the role of the transcription factor NF-κB in ageing and age-related diseases. While NF-κB is crucial for cellular survival during acute infections, a shift in the intracellular redox state during ageing could cause chronic activation of NF-κB, leading to prolonged inflammatory responses that contribute to many age-related chronic diseases. Thus, oxidative stress and NF-κB activation could be beneficial when transiently activated during infections but harmful when persistently elevated. Exploring the similarities and differences between acute and prolonged oxidative stress can offer valuable insights into the pathogenesis of many age-related diseases. Excessive ROS can cause oxidative damage, leading to cell cycle arrest, apoptosis, and various pathological conditions, including cancer and neurodegenerative diseases. Understanding the dual roles of ROS in cell cycle regulation is crucial for developing therapeutic strategies aimed at modulating ROS levels and mitigating their harmful effects.

Therapeutic strategies targeting oxidative stress include antioxidants, redox-active compounds, and modulators of redox-sensitive signaling pathways to mitigate damage and improve patient outcomes. One therapeutic strategy involves the direct exogenous intake of antioxidants. However, due to the rapid reactivity of oxidants, external scavengers often fall short compared to the numerous endogenous molecules already present in biological systems [138]. Enzymatic antioxidants like SOD and GPx are more efficient at removing ROS compared to small molecule antioxidants. SOD mimetics and GPx mimics, which replicate these enzymes’ activities, have been extensively studied for their potential to mitigate oxidative stress [138]. SOD mimetics, synthetic compounds replicating SOD activity, show significant promise in therapy. For example, manganese porphyrins shield healthy cells from radiation-induced damage in mouse models [139]. Administration of GC4419 reduced radiation-induced oral mucositis in head and neck cancer patients [140], while salen-Mn complexes have shown neuroprotective effects in neurodegenerative disease models [141]. GPx mimetics, like Ebselen and ALT-2074, reduce hydrogen peroxide and lipid peroxides by utilizing glutathione as a substrate. Ebselen has shown efficacy in reducing ischemia-reperfusion injury in stroke and myocardial infarction [142,143] and holds promise for neuroprotection and anti-inflammatory effects in neurodegenerative diseases [144].

Levels of GSH, a critical intracellular antioxidant, can be enhanced using GSH esters (monomethyl, monoethyl, diethyl, and isopropyl) to improve cellular uptake or by supplementing NAC, a GSH precursor used in treating acetaminophen overdose, chronic bronchitis, and some psychiatric disorders [138]. NOX inhibitors (diphenyleneiodonium, Ebselen, CYR5099, Apocynin, GKT137831) reduce ROS production and protect tissues, showing potential in cardiovascular diseases, neurodegenerative disorders, and cancer [138,145,146].

### ROS modulation in cancer therapy

In cancer treatment, ROS modulation is a double-edged sword. While high levels of ROS can induce apoptosis in cancer cells, excessive oxidative stress can also contribute to cancer progression and resistance to therapy. Pro-oxidant cancer therapy aims to selectively increase ROS levels in cancer cells, exploiting their already elevated oxidative stress. Chemotherapeutic agents such as doxorubicin and cisplatin generate ROS as part of their cytotoxic mechanism, leading to DNA damage, cell cycle dysregulation, and apoptosis in cancer cells. Combining ROS-inducing agents with other therapies can improve outcomes. This approach is particularly effective in tumors resistant to conventional therapies [147]. For example, apigenin selectively induces cell cycle arrest in cancer cells, inhibits the upregulation of thymidylate synthase, enhancing the efficacy of 5-FU in colorectal cancer cells [148]. It also improves the effectiveness of paclitaxel in HeLa cells through induction of ROS production [149]. Similarly, quercetin and cucurbitacin B also increase ROS production, enhancing the efficacy of paclitaxel in prostate cancer treatment and cisplatin in ovarian cancer therapy [150,151]. Interestingly, quercetin exhibits dose-dependent effects: at low doses, it acts as an antioxidant, reducing oxidative stress and offering protective benefits in conditions like diabetes and neurodegenerative diseases [152,153].

Moreover, compounds like β-lapachone and elesclomol, specifically increase ROS production and induce cell cycle arrest and apoptosis in cancer cells in preclinical models by targeting metabolic pathways unique to these cells [154,155]. However, out of 20 examined clinical trials (clinicaltrials.gov, selected filters: Intervention/treatment: elesclomol or β-lapachone), only one with elesclomol has posted results, which were evaluated as too insignificant for further investigation [156]. Additionally, elesclomol's effectiveness as an anticancer agent relies on oxygen and OXPHOS, making it potentially ineffective in glycolytic tumors [157,158].

### Therapeutic applications of antioxidants

The therapeutic potential of antioxidants has been explored in various contexts, particularly in cancer treatment. Several well-known antioxidants, such as L-ascorbic acid (vitamin C) and α-tocopherol (vitamin E), along with essential enzyme cofactors like selenium and riboflavin, play crucial roles in maintaining redox balance, and their potential therapeutic benefits are explored through various ongoing clinical studies and trials.

Vitamin C, a potent reducing agent, participates in neutralizing free radicals but has been shown to exhibit pro-oxidant effects under certain conditions, particularly at high doses [159]. This dual function is exploited in cancer therapy, where high-dose intravenous vitamin C, combined with chemotherapy, has demonstrated enhanced tumor shrinkage in mouse models and reduced side effects in ovarian cancer patients [160]. A combination of vitamin C and chemotherapy or radiotherapy is actively investigated in several clinical trials (NCT03508726, NCT04634227, NCT01852890, NCT03541486, NCT02905578, NCT06083454). Its pro-oxidant mechanism involves the disruption of intracellular iron metabolism and sensitization of cancer cells to oxidative damage [160,161]. Similarly, vitamin E, particularly in its delta-tocotrienol form, has shown promise in inducing apoptosis in neoplastic cells and enhancing chemopreventive outcomes in pancreatic cancer patients [162]. Astaxanthin, a carotenoid with strong antioxidant properties, has shown beneficial effects in Polycystic Ovary Syndrome (PCOS) by enhancing serum Total Antioxidant Capacity and activating the Nrf2 pathway in granulosa cells [163].

The meta-analyses consistently show that green tea consumption is associated with a reduced risk of several types of cancers, particularly those related to reproductive organs, the lung, non-Hodgkin's lymphoma, and oral cancer [164]. Some studies, such as those assessing green tea's impact on cardiovascular risk factors and glycemic control, show both significant and non-significant outcomes, reflecting its complex role in health [165,166]. In the context of HPV-related cervical disease, green tea extract did not significantly impact disease progression [167]. Despite mixed results, green tea is being extensively investigated as evidenced by a search on ClinicalTrials.gov for active studies using ‘green tea’ as an intervention/treatment, which yielded 132 results as of May 2024.

Melatonin, another potent antioxidant, has shown mixed results, with significant benefits observed in advanced cancer patients but not in early-stage disease [168]. Curcumin, a compound found in turmeric, shows significant therapeutic potential due to its anti-inflammatory, antioxidant, and anticancer properties [169]. Studies have demonstrated its ability to modulate inflammatory pathways and enhance the efficacy of cancer treatments. For instance, oral curcumin reduced the severity of radiation dermatitis in breast cancer patients [170]. However, its clinical application is hampered by poor bioavailability, which results in low plasma and tissue levels, thereby limiting its efficacy [171]. Despite promising findings, some clinical trials report mixed results; for example, adding curcumin to chemoradiotherapy did not improve complete response rates in patients with metastatic prostate cancer or locally advanced rectal cancer [172,173]. These inconsistent outcomes highlight the need for improved curcumin formulations with better bioavailability and further research to determine optimal dosages that ensure safety and efficacy.

Cancer cells often have upregulated antioxidant systems to manage their higher ROS levels. Inhibiting these antioxidant defenses can disrupt the redox balance, leading to oxidative stress cell cycle arrest and cell death. This strategy targets the cancer cells’ reliance on antioxidant systems to survive in a high-ROS environment. Agents like buthionine sulfoximine, which depletes GSH, enhance the efficacy of ROS-inducing therapies by preventing cancer cells from neutralizing ROS [147]. Inhibitors of enzymes such as SOD and catalase can also reduce the capacity of cancer cells to neutralize ROS.

Given the mixed outcomes and the potential for antioxidants to both aid and hinder therapy, their use must be tailored to individual patient profiles and specific treatment contexts. Advances in personalized medicine, including the identification of biomarkers for oxidative stress and antioxidant capacity, will be crucial in optimizing antioxidant use in therapy. Large-scale, well-designed clinical trials are needed to provide definitive evidence of redox treatment efficacy and safety.

## Conclusion

**Figure 4.**
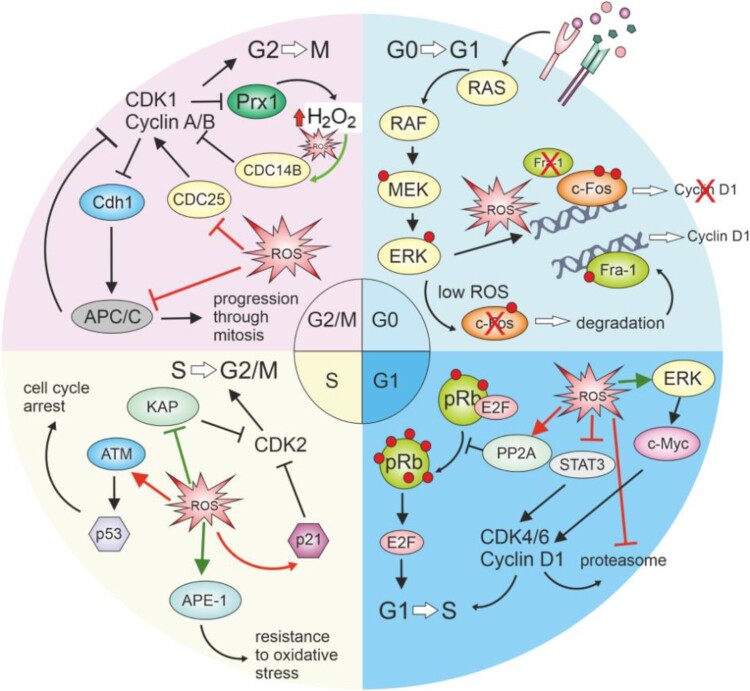
Redox-mediated regulation of cell cycle phases. Increased ROS during the transition from G0 to G1 signaling can cause continuous ERK signaling and halt c-fos degradation, subsequently preventing the access of the transcription factor Fra-1 to chromatin, which is necessary for the transcription of cyclin D. In the G1 phase, ROS can cause the cell to halt the cycle due to the inhibition of pRB hyperphosphorylation, which, in this state, will not allow the release of E2F transcription factors necessary for progression to the S phase. ROS can affect the signaling function of STAT3 and inhibit cycle progression. ROS was suggested to prevent the breakdown of cyclin D by inhibiting the proteasome. However, positive effects have also been described, where ROS mediate c-myc activation through ERK signaling and upregulation of cyclin D. In the S phase, ROS can contribute to cellular resistance to oxidative stress through APE-1 and enhancing the repair capacity of the cell, while also mediating passage through the S phase by inhibiting KAP, thus enabling phosphorylation of Cdk2. During the S phase, ROS can also contribute to the cell's decision to stop or exit the cell cycle by damaging cell structures and activating the checkpoint through ATM and p53, or they can activate p21, which subsequently inhibits the activity of CDK2. In the G2/M phase, ROS exert an inhibitory effect on CDC25 and APC/C, preventing mitosis progression. Still, it is assumed that a lower amount of ROS is necessary for the inhibition of CDC14B, which subsequently leads to the activation of APC. Pathways indicated in red color show the inhibitory effect of ROS on cell cycle progression, while green lines represent pathways supporting its progression.

Maintaining redox homeostasis within the cell is critical, as its disturbance can lead to dysregulated proliferation or cell death. Experimental studies demonstrate the possibility for exogenous control of this homeostasis, offering potential therapeutic avenues for diseases associated with heightened oxidative stress. However, leveraging this knowledge in therapy requires a thorough understanding and prediction of the cell's response to oxidative stress. The precise role of ROS in cell cycle regulation remains uncertain. While some studies highlight the necessity of protein oxidation for its progression, others emphasize undesirable inhibitory effects (Figure 4). Redox regulation of proteins involved in the cell cycle, as detailed in the Table 2, shows how reactive cysteines can influence their function. For instance, modifications such as disulfide bonds and sulfenic acid formation can activate or inactivate key proteins, impacting cell cycle progression. It is evident that the concentration of oxidants within the cell has a key role, but determining what concentrations are considered favorable or damaging for cell signaling remains unclear. To employ redox signaling in clinical research, it is imperative to conduct further research that accurately describes the effects of ROS based on the concentration, cell phase, and cell type. Only through comprehensive investigations can we gather the necessary insights to influence cell fate through redox signaling.

**Table 2.** Identified reactive cysteine modifications in cell cycle regulators.

Protein	Reactive cysteine	Modification type	Effect	References
KEAP-1	Cys151	Disulfide bond	Nrf2 activation	[53]
IKK	Cys179	Glutathionylation	IKK inhibition	[20]
p50	Cys62	Glutathionylation	p50 inhibition	[56]
PTP1B	Cys215	Sulfenic acid	PTP1B inactivation	[77]
PTEN	Cys71, Cys124	Disulfide bond	PTEN inactivation	[81]
Src family kinases	Cys277	Disulfide bond	Src inactivation	[84]
PAK	Cys199	Glutathionylation	PAK inactivation	[83]
Akt	Cys310	Sulfenic acid	Akt ativation	[85]
	Cys60, Cys310	Sulfonic acid	Akt accumulation in mitochondria, apoptosis	
Ask-1	multiple	Oligomerization, disulfide bonds	Ask-1 activation, apoptosis	[87]
MEKK1	Cys1238	Glutathionylation	MEKK1 inactivation	[90]
Atg4	Cys77, Cys81	Sulfenic acid	Atg4 inhibition, autophagosome formation	[91]
CDK4	Cys135	Disulfide bond with cyclin D	inhibition of cyclin D-CDK4 activity	[93]
cyclin D	Cys7/8	Disulfide bond with CDK4	inhibition of cyclin D-CDK4 activity	[93]
Stat3	multiple	Glutathionylation	decreased STAT3 regulated expression	[100]
CDK2	Cys177	Sulfenic acid	CDK2 activation	[104]
APE1	multiple	Sulfenic acid	impaired function	[109]
p53	multiple	Various	both, positive and negative effects on DNA binding	[122,123]
Cdc25	Cys377, Cys330	Disulfide bond	inactivation	[128,129]
APC	coordinating cysteines	Disulfide bond	APC inable to bind Ubc4	[6]
Aurora A	Cys290	Unclear	Aurora A inactivation	[24]

## Author statement

**Viktoria Mackova** participated in writing of manuscript, design of figures and table preparations.

**Martina Raudenska** participated in writing and supervision of manuscript and created all figures in graphical software. **Hana Holcova Polanska** and **Milan Jakubek** participated in writing and supervision of manuscript. **Michal Masarik** design concept of manuscript and participated in manuscript supervision.

## Data Availability

Data sharing is not applicable to this article as no new data were created or analyzed in this study.
